# Evaluating surface coatings to reduce bone cement adhesion to point of care 3D printed molds in the intraoperative setting

**DOI:** 10.1186/s41205-022-00156-6

**Published:** 2022-08-12

**Authors:** Brian Beitler, Gregory R. Roytman, Grace Parmer, Steven M. Tommasini, Daniel H. Wiznia

**Affiliations:** 1 Department of Orthopaedics and Rehabilitation, Yale School of Medicine, Yale University, 800 Howard Ave 1st Floor, New Haven, CT 06519 USA; 2 Yale Center for Medical Informatics, Yale School of Medicine, Yale University, 300 George Street, Ste 501, New Haven, CT 06511 USA; 3 VA Connecticut Healthcare System, Veterans Health Administration, 950 Campbell Ave, West Haven, CT 06516 USA; 4 Department of Biomedical Engineering, Yale School of Engineering and Applied Science, Yale University, 17 Hillhouse Ave, New Haven, CT 06511 USA; 5 Department of Mechanical Engineering & Materials Science, Yale School of Engineering and Applied Science, Yale University, 17 Hillhouse Ave, New Haven, CT 06511 USA

**Keywords:** 3D printing, Bone cement, Orthopaedic surgery, Personalized medicine

## Abstract

**Background:**

Polymethyl methacrylate, or “bone cement,” can be used intraoperatively to replace damaged or diseased bone and to deliver local antibiotics. 3D printed molds allow surgeons to form personalized and custom shapes with bone cement. One factor hindering the clinical utility of anatomically accurate 3D printed molds is that cured bone cement can be difficult to remove due to the strong adhesion between the mold and the bone cement. One way to reduce the adhesion between the 3D printed mold and the cured bone cement is with the use of a surface coating, such as a lubricant. This study sought to determine the optimal surface coating to prevent bone cement adhesion to 3D printed molds that could be utilized within a sterile operating room environment.

**Methods:**

Hemispheric molds were 3D printed using a stereolithography printer. The molds were coated with four sterile surface coatings available in most operating theatres (light mineral oil, bacitracin ointment, lubricating jelly, and ultrasound transmission gel). Polymethyl methacrylate with tobramycin antibiotic was mixed and poured into the molds. The amount of force needed to “push out” the cured bone cement from the molds was measured to determine the efficacy of each surface coating. Tukey’s multiple comparison test was performed to compare the results of the pushout test.

**Results:**

The average pushout force for the surface coatings, in increasing order, were as follows (mean ± standard deviation) --- bacitracin ointment: 9.10 ± 6.68 N, mineral oil: 104.93 ± 69.92 N, lubricating jelly: 147.76 ± 63.77 N, control group: 339.31 ± 305.20 N, ultrasound transmission gel 474.11 ± 94.77 N. Only the bacitracin ointment required significantly less pushout force than the control (*p* = 0.0123).

**Conclusions:**

The bacitracin ointment was the most effective surface coating, allowing the bone cement to be pushed out of the mold using the least amount of force. In addition, the low standard deviation speaks to the reliability of the bacitracin ointment to reduce mold adhesion compared to the other surface coatings. Given its efficacy as well as its ubiquitous presence in the hospital operating room setting, bacitracin ointment is an excellent choice to prevent adhesion between bone cement and 3D printed molds intraoperatively.

## Introduction

3D printing allows for the manufacturing of complex and patient specific geometries, allowing for personalized treatment [1–4]. 3D printing is being increasingly utilized by individual practitioners at the point of care (PoC) [5–10]. An increase in the accessibility of 3D printers with biocompatible printing capabilities has led to an increase in PoC intraoperative uses, such as the creation of molds to create patient-specific implants within the operating room.

One field where there is a potential for PoC 3D printed molds is orthopaedic surgery, where 3D printing is already a useful tool [11–13]. In addition, there are numerous situations where polymethyl methacrylate (PMMA), referred to in orthopaedics as “bone cement,” is used as a replacement or augmentation for resected or diseased bone and to deliver local antibiotics [14–16]. Bone cement used in this way spans orthopaedic subspecialties, from musculoskeletal oncology where bone cement is used to fill lytic lesions to spinal surgery where bone cement is used to reinforce osteoporotic, stress-fractured vertebrae (vertebroplasty). In most cases, the bone cement is shaped by hand, inserted through a syringe, or hand-pressed into the space it is reinforcing. In other cases, makeshift molds such as chest tubes or bulb syringes are used to create desired shapes [15, 16]. PoC 3D printed molds offer another, more precise method for the creation of desired bone cement geometries.

**Fig. 1:**
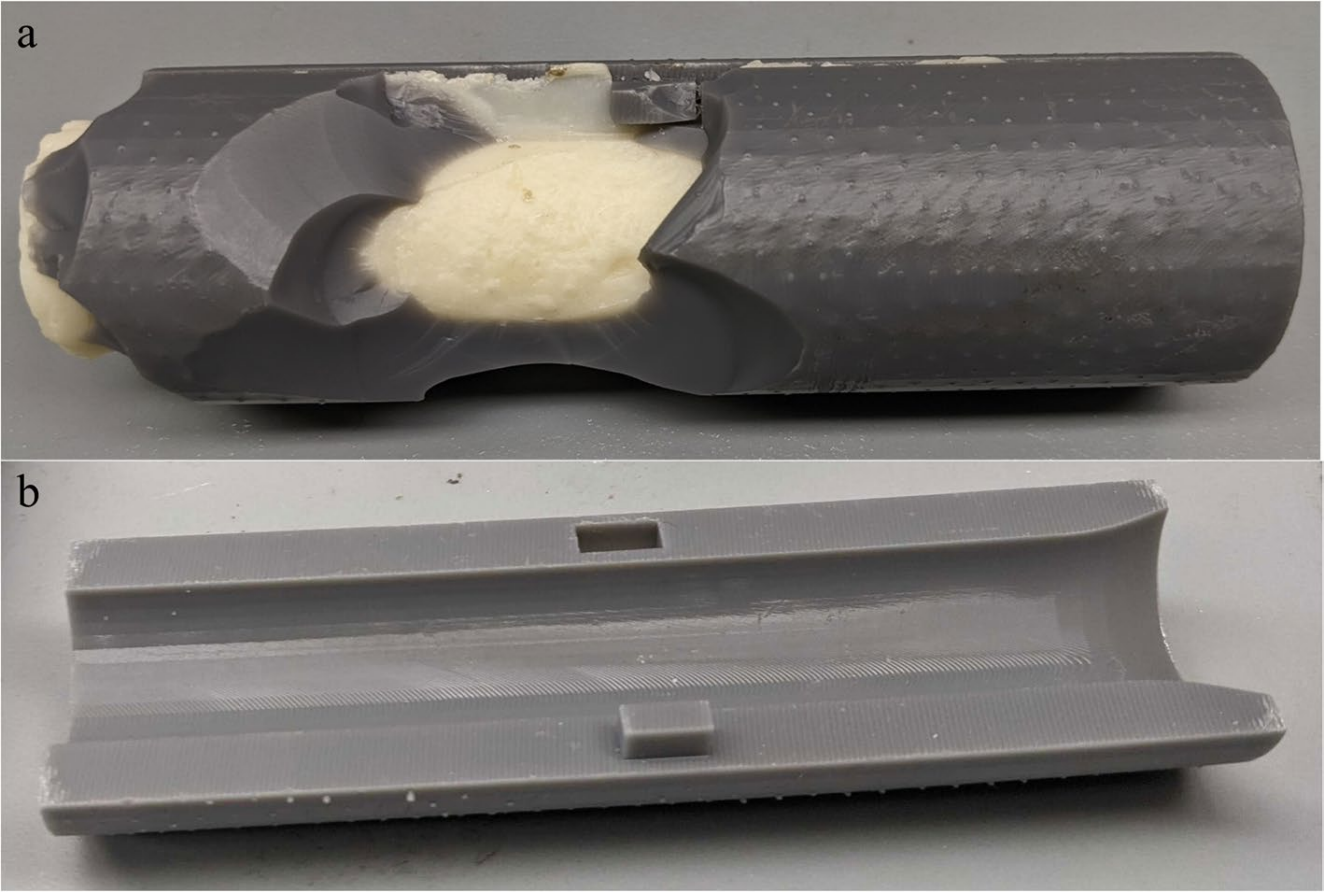
An example of the result of trying to remove bone cement that has strongly adhered to a 3D printed mold (**a**). In this example, bone cement was poured into a cylindrical mold composed of two half-cylinders printed using Formlabs (Somerville, MA) Grey V4 resin (**b**). After curing, the bone cement was too strongly adhered to the mold to be removed. The damage seen above (**a**) is the result of a failed attempt to chisel the mold off of the bone cement

One challenge to overcome with PoC 3D printed molds (and molds in general) for bone cement, however, is that bone cement can become strongly adherent to the surface of the mold during the curing process of the bone cement [15, 16]. This can make the form difficult to remove from the cement without damage (Fig. 1). Surface coatings, which act as a potential barrier to reduce adhesion, present a simple solution to this challenge. Surface coatings such as mineral oil have actually already been used to prevent bone cement adhesion to surfaces, with variable success [16–18]. Optimally, the surface coating used would be one that is sterile, biocompatible, and, if possible, readily available in the operating room. Furthermore, the surface coating should not interfere with the curing process. This study explored four surface coatings that met these criteria in order to evaluate their ability to prevent bone cement adhesion to a 3D printed mold.

## Methods

**Fig. 2:**
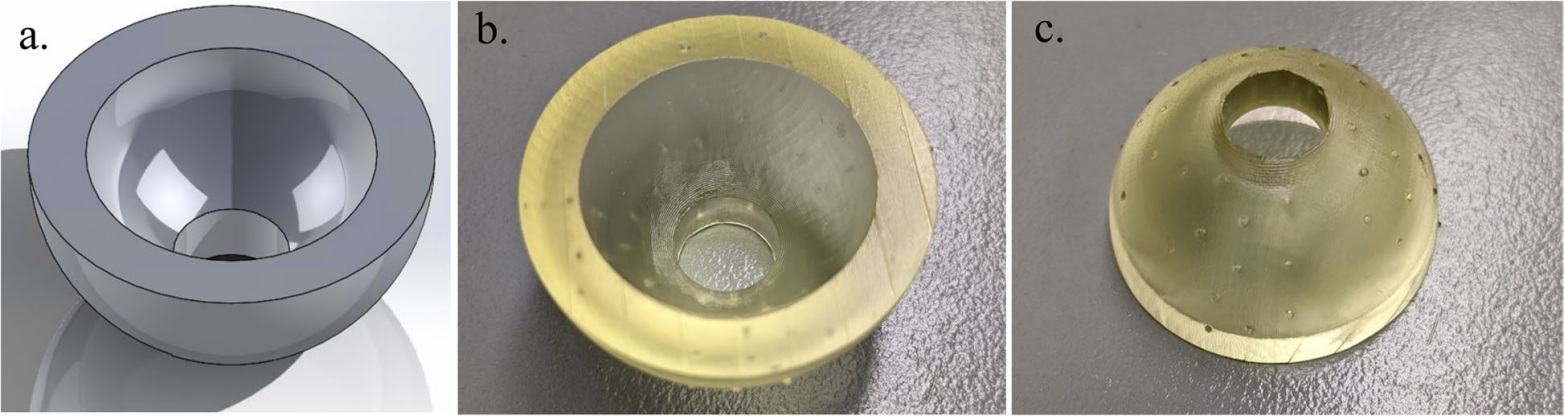
The SolidWorks model of the hemispherical mold (**a**). Concave surface of mold (**b**). Back of mold (**c**). The inner diameter of the mold is 2.5 cm with a height of 1.25 cm, and the outer diameter is 3.5 cm with a height of 1.75 cm

**Fig. 3:**
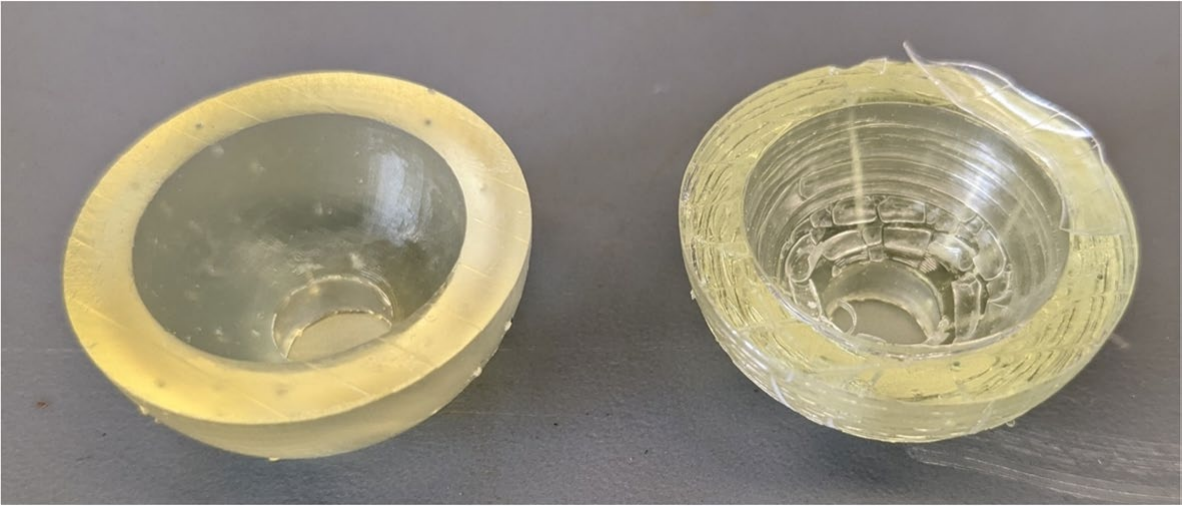
Normal mold (left) next to a mold deformed by the autoclave cycle due to remaining IPA from the wash step that did not evaporate before the post-cure step (right)

**Fig. 4:**
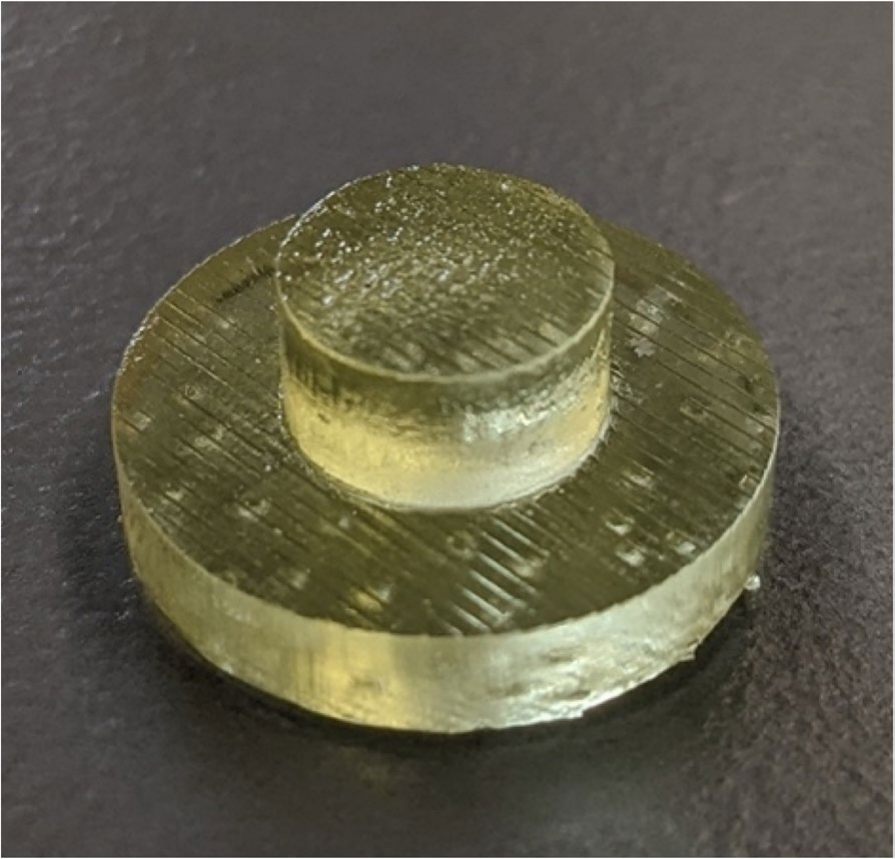
One of the plugs used to fill the hole in the base of the hemispherical mold shown in Fig. 2

Hemispherical molds with an inner diameter of 2.5 cm and an outer diameter of 3.5 cm were designed in SOLIDWORKS (Dassault Systèmes, Vélizy-Villacoublay, France) (Fig. 2a**)**. The molds were designed with a 1 cm diameter hole at the bottom to allow for a probe connected to a force sensor to push out the bone cement during experimentation. The molds were printed using a Form 3BL (Formlabs, Somerville, MA) stereolithography printer using the biocompatible Biomed Amber resin with 0.100 mm z-axis resolution (Fig. 2b and c). A desktop inverted vat photopolymerization 3D printer (the Form 3BL) was used, as this type of printer is capable of printing with high enough resolution for medical applications and can print using biocompatible materials [8]. In addition to being biocompatible, the high melting point of this material allowed it to avoid issues that have faced other materials used as bone cement molds, as the methyl -methacrylate hardens in an exothermic reaction [16]. After printing, the molds were washed with isopropyl alcohol (IPA) for 20 minutes, “post-cured” with UV light at 70C for 30 minutes, and autoclaved at 134C for 20 minutes per the official resin documentation [19]. In order to ensure that the IPA adequately evaporated before post-curing, the molds were left to sit for 30 minutes, and then underwent a 30 minute “preheat” cycle at 60C after the wash. This was to prevent the catastrophic deformation that can occur in the autoclave if the IPA is not allowed to fully evaporate **(**Fig. 3**)**. In addition to the molds, plugs for the holes at the base of the molds were designed and printed (Fig. 4).

Four different sterile surface coatings available for use in the operating room were tested: Muri-Lube light mineral oil (Fresenius Kabi, Bad Homburg, Germany), bacitracin zinc ointment (Stratus Pharmaceuticals, Miami, FL), MediChoice lubricating jelly (Owens and Minor, Richmond, VA), and ultrasound transmission gel (Parker Laboratories, Fairfield, NJ). The mineral oil was in liquid form, and the three other surface coatings were in gel form. The bacitracin ointment has a petroleum jelly base, and the lubricating jelly and ultrasound gel were water-based. Thus, there were two oil-based and two water-based surface coatings tested. To apply the mineral oil, the molds were submerged in the liquid for 5 min. To apply the gels, the molds were coated with a thin layer with a gloved hand. The exact amount gel applied varied slightly between cups, but could be described as “the minimum amount required to coat the entire surface.” This approach was chosen to reflect what could be done easily in the operative setting. After a given mold was lubricated, the plug was inserted into the base of the mold.

**Fig. 5:**
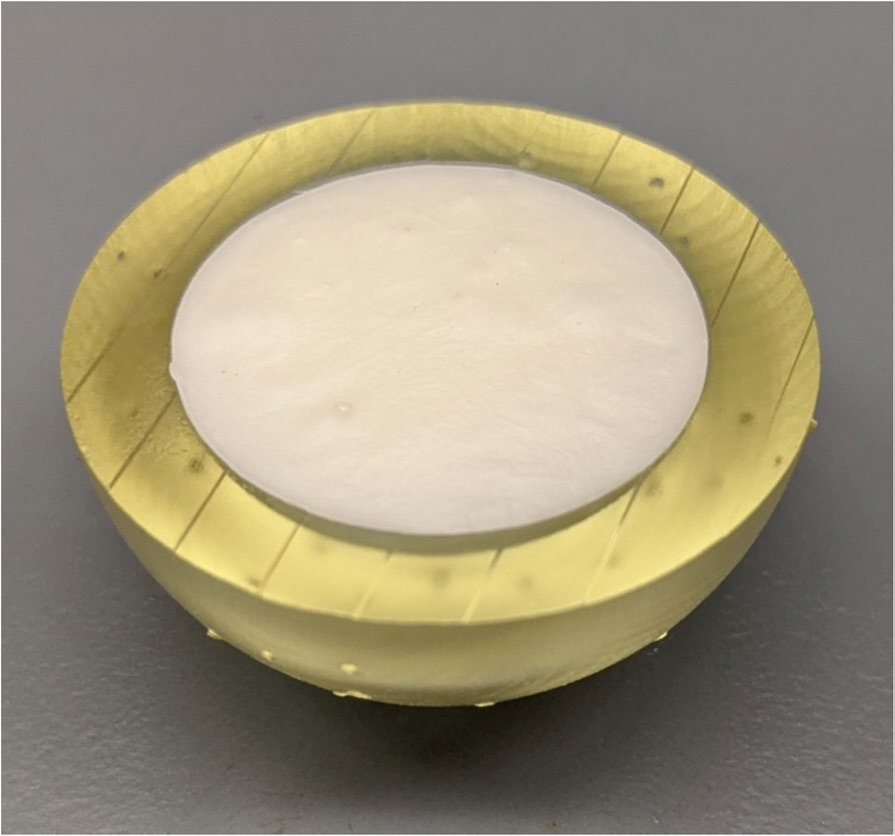
A hemispherical mold filled with bone cement

Simplex P with Tobramycin antibiotic bone cement (Stryker Corporation, Mahwah NJ) was chosen due to its low viscosity, allowing it to be poured, which ensured an even distribution within the mold. The bone cement was mixed per the instructions and poured into each mold. Only enough cement to pour into four molds was prepared at a time so that cement was poured into each mold at roughly the same time in the cement cure process (within 30 seconds of each other) for intra and inter batch consistency. If a mold was not filled within this 30-second window, it was discarded. A filled mold can be seen in Fig. 5. This method of discarding molds if they were not filled within the allotted time-frame led to unequal sample sizes. The sample sizes were as follows: Control group (*n* = 6), bacitracin ointment (*n* = 6), mineral oil (*n* = 7), lubricating jelly (*n* = 6), ultrasound transmission gel (*n* = 3).

While the bone cement was curing, the plugs were rotated every minute to ensure that the cement did not adhere to the plugs. This allowed the plugs to be removed once the cement hardened. The bone cement was allowed to cure for 20 minutes, which was sufficient time for it to fully cure at room temperature (22C), per the bone cement manufacturer’s protocol [20]. Once the cement was cured, the plug was removed from the base of the mold.

**Fig. 6:**
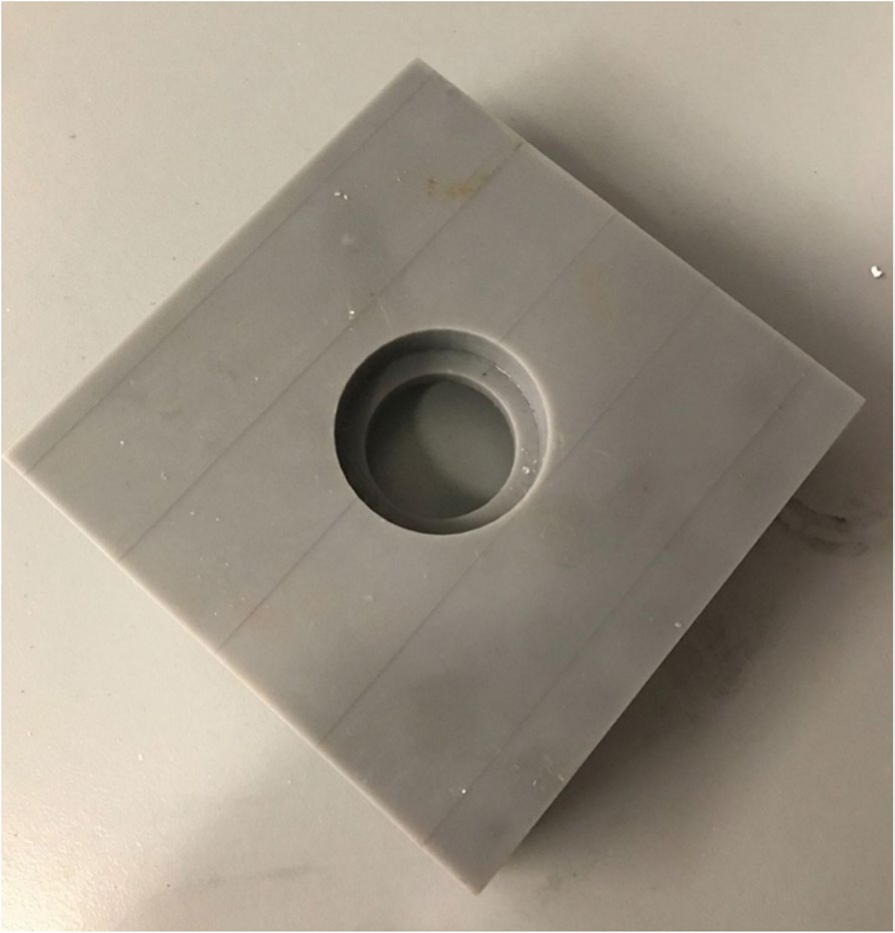
The 3D printed jig used to position the filled molds for testing with the Instron

**Fig. 7:**
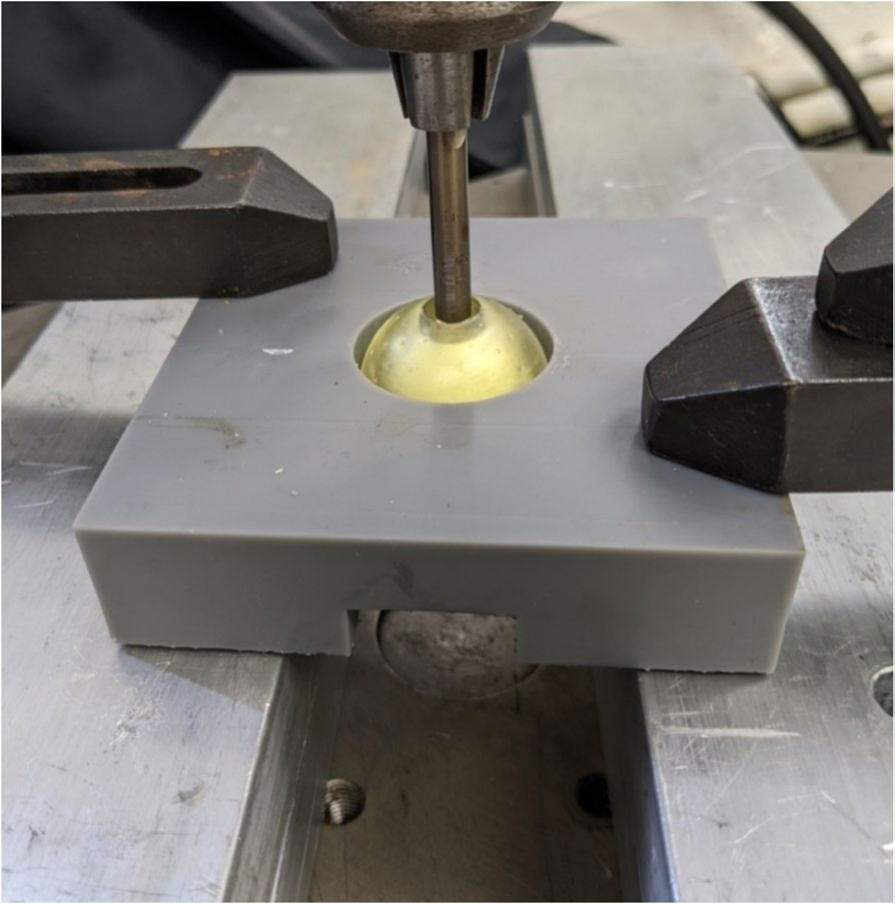
The full setup of jig, mold with cement, and Instron probe

In order to determine the efficacy of the different lubricants, the force required to push out the bone cement from each mold was measured. A custom 3D printed jig was used to hold the molds so the cement could be pushed out (Fig. 6). Molds were placed in the jig, and positioned so that the loading apparatus aligned with the hole in the base of the mold. Tests were conducted with a deflection rate of 0.05 mm/sec using a servohydraulic testing machine (Instron model 8874; Instron Corp., Norwood, MA, USA). The full loading setup is shown in Fig. 7. Load and displacement data were captured at a rate of 200 Hz. Tests were ended when the hardened piece of bone cement fell out of the mold.

## Results

**Fig. 8:**
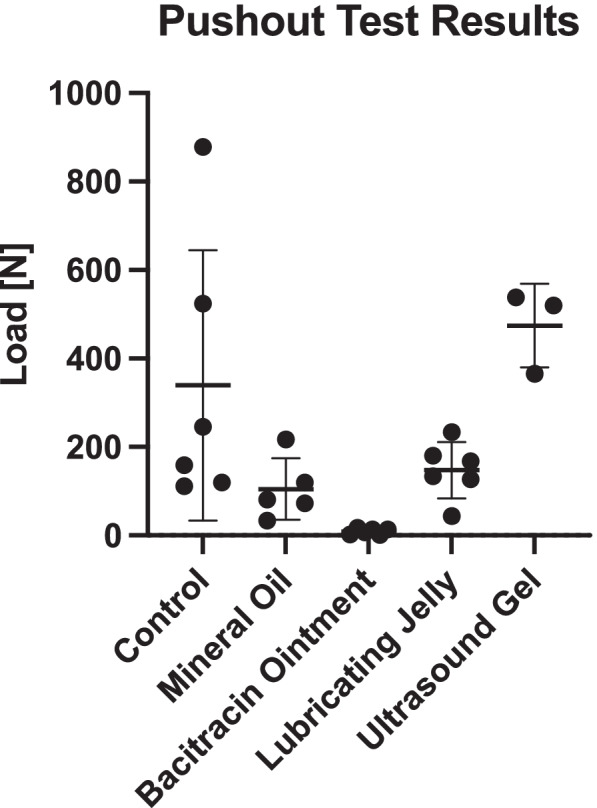
This graph shows the average maximum load needed for each lubricant to push the hardened cement out of the mold during Test 2

The average pushout force for the surface coatings, in increasing order, were as follows (mean ± standard deviation (sample size)) --- bacitracin ointment: 9.10 ± 6.68 N (*n* = 6), mineral oil: 104.93 ± 69.92 N (*n* = 7), lubricating jelly: 147.76 ± 63.77 N (*n* = 6), control group: 339.31 ± 305.20 (*n* = 6), ultrasound transmission gel N 474.11 ± 94.77 N (*n* = 3) (Fig. 8**)**. Only the bacitracin ointment required significantly less pushout force than the control (*p* = 0.0124). The individual data points can be found in Table 1.

**Table 1:** Complete Pushout Test Data

	Surface Coating				
	Control	Mineral Oil	Bacitracin Ointment	Lubricating Jelly	Ultrasound Transmission Gel
**Pushout Force (N)**	245.23	80.95	7.03	43.53	537.69
	111.59	73.22	16.93	168.15	519.46
	158.34	33.46	13.7	133.89	365.19
	877.58	982.84	1.87	180.61	
	523.92	939.59	13.85	126.92	
	119.18	217.41	1.24	233.46	
		119.59			

## Discussion

The goal of this study was to identify which surface coating available in the surgical setting would be most effective at preventing adhesion between bone cement and a 3D printed mold. To accomplish this, the force required to remove bone cement from identical molds with various surface coating was measured and compared. The bacitracin ointment was the most effective and reliable lubricant for preventing bone cement-mold adhesion and the only lubricant with a pushout force significantly lower than the control group (*p* = 0.0124). Not only did the bacitracin group have the lowest average pushout force, it also had the lowest standard deviation of any group, an indication of its reliability as a surface coating. Though not included in the data due to inconsistent experimental protocols, it should be noted that during pilot studies the bacitracin ointment was sometimes so effective the bone cement would sometimes simply fall out of the mold before testing. While the other surface coatings had lower average pushout forces than the controls, their large standard deviations make them inconsistent choices for delicately casted parts.

Notably, the most effective lubricants were all oil based. There are multiple potential explanations for this. The first explanation may be how the water-based lubricants interact with the mold and bone cement surfaces at the mold-cement interface, as it is known that water and oil interact differently with the hydrophobic PMMA [18, 21]. The second is that the water component of the water-based lubricants may have evaporated or partially evaporated during the bone cement curing process, as the curing process is exothermic. Adding plausibility to this hypothesis is the observation that the water-based lubricant coatings dried out if they were applied more than 10 minutes before the bone cement was poured. Due to this fact, all lubricants were applied within 5 minutes of pouring the bone cement. This added layer of logistical complication is further reason that water-based lubricants (e.g. the lubricating jelly) would make suboptimal intraoperative mold lubricants. Further considerations based on observations from testing can be found in Table 2.

**Table 2:** Considerations for each surface coating

Surface Coating	Considerations
Mineral Oil	Has a tendency to pool in depressed area. This may be an issue for some mold geometries. The submersion application technique may be difficult for larger molds. Alternate application techniques may be required.
Bacitracin ointment	Not appropriate for patients with a bacitracin allergy.
Lubricating jelly	The water component evaporates, so should be applied shortly (< 5 minutes) before pouring the bone cement.
Ultrasound transmission gel	Same as lubricating jelly.

Of the oil-based lubricants, the bacitracin ointment proved to be the most effective. Though there are multiple reasons this might be, the most likely are related to its high viscosity. This high viscosity likely leads to a thicker barrier layer between the bone cement and the mold compared to the liquid oil lubricants. Furthermore, this higher viscosity makes the bacitracin ointment more difficult for the cement to displace the bacitracin coating from the mold surface during the casting process. Interestingly, despite examples in the literature of mineral oil being used to prevent bone cement surface adhesion, the mineral oil did not appear to be particularly effective at preventing adhesion to the 3D printed molds used in this study [16–18]. This may be due to the fact that in the cases described in the literature, the mineral oil was used to coat extremely smooth plastic surfaces (e.g. chest tubes) as opposed to the textured surfaces of 3D printed molds to which bone cement adheres more strongly. The extent that surface texture affects bone cement adhesion to 3D printed molds is a topic which should be explored in the future.

Given the current small body of literature on this topic, there were observations made during the study design process that bear discussing. The first set of observations pertains to the selection of bone cement used for molds. Initially, the bone cement selected for these experiments was a high viscosity cement. However, it proved suboptimal for multiple reasons. The first was that it cured relatively quickly, meaning that its physical properties were different when filling each mold in a given batch. Second, it filled the molds inconsistently, often leaving air pockets without bone cement, and required a significant amount of manual manipulation. Third, when adding additional cement, the cement had a tendency to layer on itself instead of forming one homogeneous mass. This issue was particularly problematic when cement had been further along in the curing process, even within 2 min of mixing. These issues also meant that filling the molds with high viscosity cement took significantly longer, compounding the viscosity-related issues described above. This led to the observation that the time between cement preparation and addition to the mold appeared to have a large effect on the pushout test results. Longer times (on the order of 1–2 minutes) between mixing the cement and adding it to the mold lead to significantly lower forces needed to push the hardened cement out when using the high viscosity cement. This relationship seemed to apply to the low viscosity cement as well, though on a longer timescale (> 2 minutes). In summary, low viscosity bone cement appears to be optimal for casting compared to high viscosity bone cement.

### Limitations

This study has a few limitations. First, this study examined the efficacy of surface coatings on 3D printed molds constructed using a single type of resin with a single set of printer settings and printed with a single orientation. Thus, though one surface coating clearly performed better than the others in this experiment, further work needs to look at the generalizability of the results. In particular, the use of surface coatings to prevent adhesion between bone cement and molds made from other types of resins, especially those with other physical properties, requires more exploration. A second limitation of this study is the relatively small sample sizes. Thus, while large differences in surface coating performance could be found, larger sample sizes are needed to find subtler performance differences. Third, this study did not look at other methods of preventing bone cement adhesion such as making physical alterations to the mold surface itself (e.g. sanding the mold).

## Conclusions

Of the surface coatings tested, including a range of oil-based and water-soluble options, we found that bacitracin ointment was the most reliable and effective. Given bacitracin ointment’s efficacy and ubiquity in the hospital operating theatre, we recommend bacitracin ointment as a preferred surface coating. Prior to using bacitracin ointment, surgeons should confirm that the patient does not have a bacitracin allergy. Further investigation should explore factors which affect mold performance such as mold geometry. Furthermore, variables related to stereolithography printers should be explored, such as different biocompatible resins and adjustable printer settings such as print orientation and z-axis resolution. Ultimately, the authors hope that this work will aid physicians working to improve patient care through personalized medicine.

## Data Availability

The datasets used and analyzed during the current study are available from the corresponding author on reasonable request.

## Abbreviations

PoC: Point of Care
PMMA: Polymethyl Methacrylate
IPA: Isopropyl Alcohol
